# Co-Occurrence of 35 Mycotoxins: A Seven-Year Survey of Corn Grain and Corn Silage in the United States

**DOI:** 10.3390/toxins13080516

**Published:** 2021-07-23

**Authors:** Alexandra C. Weaver, Daniel M. Weaver, Nicholas Adams, Alexandros Yiannikouris

**Affiliations:** 1 Alltech Inc., 3031 Catnip Hill Road, Nicholasville, KY 40356, USA; ayiannikouris@alltech.com; 2 Independent Researcher, Orrington, ME 04474, USA; dmw1121@gmail.com; 3 Alltech UK, Stamford PE9 1TZ, UK; nadams@alltech.com

**Keywords:** co-occurrence, corn, deoxynivalenol, fusaric acid, *Fusarium*, monitoring, maize, mycotoxins, silage

## Abstract

Mycotoxins contaminate crops worldwide and play a role in animal health and performance. Multiple mycotoxins may co-occur which may increase the impact on the animal. To assess the multiple mycotoxin profile of corn (*Zea mays*), we conducted a 7-year survey of new crop corn grain and silage in the United States. A total of 711 grain and 1117 silage samples were collected between 2013 and 2019 and analyzed for the simultaneous presence of 35 mycotoxins using ultra-performance liquid chromatography–tandem mass spectrometry. The measured mean number of mycotoxins per sample were 4.8 (grain) and 5.2 (silage), ranging from 0 to 13. Fusaric acid (FA) was most frequently detected in 78.1 and 93.8% of grains and silages, respectively, followed by deoxynivalenol (DON) in 75.7 and 88.2% of samples. Fumonisin B1 (FB1), fumonisin B2 and 15-acetyl-deoxynivalenol (15ADON) followed. The greatest (*p* < 0.05) co-occurrence was between FA and DON in 59.1% of grains and 82.7% of silages, followed by FA with FB1, DON with 15ADON, and FA with 15ADON. Although many samples had lower mycotoxin concentrations, 1.6% (grain) and 7.9% (silage) of tested samples had DON ≥ 5000 µg/kg. Fumonisins were detected ≥ 10,000 µg/kg in 9.6 and 3.9% of grain and silage samples, respectively. Concentrations in grain varied by year for eight mycotoxin groups (*p* < 0.05), while all 10 groups showed yearly variations in silage. Our survey suggest that multiple mycotoxins frequently co-occur in corn grain and silage in the United States, and some of the more prevalent mycotoxins are those that may not be routinely analyzed (i.e., FA and 15ADON). Assessment of multiple mycotoxins should be considered when developing management programs.

## 1. Introduction

In the United States, corn (*Zea mays*) is an important grain and forage crop and is used as the primary energy ingredient in livestock feed. As a cereal, corn is subject to infection by numerous diseases including fungal diseases of the *Aspergillus*, *Fusarium* and *Penicillium* genera [1]. Colonization and growth of these fungi can lead to a variety of impacts on the crop, such as reduced yield and altered quality, as well as mycotoxin contamination [2]. Mycotoxins, or secondary metabolites produced by molds, are particularly problematic for livestock production due to capability of these compounds to modulate metabolism and microbial response including adverse effects on intestinal and immune health, reproduction, gain and efficiency [3].

The growth of molds and the production of mycotoxins is influenced by a variety of factors including climatic conditions, agronomic practices and physical handling of grains and plant material [4]. Furthermore, mycotoxins can be produced at all steps of crop and feed production, i.e., before harvest, at harvest, during feedstuff storage, throughout feed processing, during storage of complete feeds or at feed out on-farm. If mycotoxins are formed at any of these steps, decreasing their contamination levels by chemical, biochemical or physical means is difficult due to their inherent stability to temperature, pH [3,4] or other biotic detoxification approaches. Therefore, livestock are likely to consume mycotoxins which could lead to negative effects on health and performance.

Although global mycotoxin regulations tend to focus on a few key mycotoxins individually, there are many different types of mycotoxins including conjugated and emerging mycotoxins [5,6]. Additionally, crops are rarely contaminated with one mycotoxin but rather numerous mycotoxins simultaneously. A recent survey of corn silage from Flanders, Belgium indicated that 47% of samples contained five or more mycotoxins out of the 22 analyzed [5]. As animals are chronically exposed to mixtures of mycotoxins during their entire lifecycle, even lower concentrations may result in interactions that impact health, production, and efficiency [7]. Therefore, assessing the presence of multiple mycotoxins in feedstuffs and finished rations is of importance, even when those mycotoxins may be below regulatory limits.

In the past decade, numerous surveys have been published from around the world showing mycotoxin contamination profiles. However, few have focused on North American corn production, with even fewer investigating mycotoxin profiles in both corn grain and whole plant corn silage. Thus, the aim of this research was to investigate mycotoxin profiles in corn grain and corn silage harvested in the United States of America over a 7-year period from 2013 to 2019. Furthermore, all samples in this study were analyzed for 35 individual mycotoxins that included isomers, conjugated and emerging mycotoxins, to track the occurrence of multiple mycotoxins in these commodities over time and without decision bias.

## 2. Results

### 2.1. Mycotoxin Concentrations and Occurrence

#### 2.1.1. Corn Grain

Measurable concentrations of mycotoxins were detected in 98.6% of corn grain samples with 90.2% of samples containing two or more mycotoxins (Table 1). The mean number of mycotoxins per sample was 4.79 ± 2.44 with a minimum of 0 and a maximum of 12 mycotoxins detected in these samples out of the 35 tested. Only 1.4% of samples did not contain mycotoxins. The most prevalent mycotoxin group was the type B trichothecenes comprising deoxynivalenol (DON), 3-acetyl-deoxynivalenol (3ADON), 15-acetyl-deoxynvalenol (15ADON), deoxynivalenol-3-glucoside (D3G), nivalenol (NIV) and fusarenon X (FX), detected in 81.7% of samples (Table 2). The emerging mycotoxins, composed of fusaric acid (FA) and alternariol, closely followed in 78.1% of grain samples, total fumonisins (FBs) which includes fumonisin B1 (FB1), fumonisin B2 (FB2) and fumonisin B3 (FB3) were in 69.3% of samples, and zearalenone (ZEA) was detected in 21.2% of samples. Type A trichothecenes, composed of T-2 toxin (T2), HT-2 toxin (HT2), diacetoxyscirpenol and neosolaniol, was detected in 13.2% of samples. All other mycotoxin groups were detected, but in less than 10% of samples.

The most prevalent individual mycotoxin was FA in 78.1% of samples, with a mean concentration of 207 ± 460 µg/kg and median of 88.7 µg/kg (Table 3). Although the maximum detected level of FA was 6792 µg/kg, 75% of positive samples had concentrations less than 205 µg/kg. Deoxynivalenol was the second most prevalent mycotoxin in 75.7% of samples. The mean for DON was 840 ± 1790 µg/kg, the median 333 µg/kg and maximum 27,000 µg/kg, with the upper 25% of positive samples having concentrations of DON above 914 µg/kg. Other prevalent mycotoxins included FB1 in 65.7%, FB2 in 53.4%, 15ADON in 47.7% and FB3 in 41.8% of samples. Fumonisin B1 was detected in corn grain at an average value of 3800 ± 6100 µg/kg (median 1340 µg/kg; maximum 52,500 µg/kg) with 75% of positive samples having concentrations less than 4750 µg/kg. Fumonisin B2 and FB3, had means of 738 ± 1150 and 462 ± 651 µg/kg (medians 204 and 162 µg/kg; maximums 5640 and 2800 µg/kg) with 75% percentiles of 772 and 554 µg/kg, respectively. The mean of 15ADON was 91.6 ± 137 µg/kg, median 52.1 µg/kg and maximum 1170 µg/kg, with 75% quartile of 96.5 µg/kg. Of additional importance was the presence of ZEA in 21.2% of samples. Zearalenone was found at an average of 302 ± 486 µg/kg, median 115 µg/kg and maximum 2890 µg/kg. Interestingly, the top 25% of positive samples had concentrations greater than 337 µg/kg. In corn grain, all other measured mycotoxins were detected in less than 20% of samples. Only NIV and ergocryptine were not detected in the 711 corn grain samples analyzed.

#### 2.1.2. Corn Silage

Mycotoxins were detected in 99.6% of corn silage samples, with 96.8% of samples containing two or more mycotoxins (Table 1). Samples contained from 0 to 13 mycotoxins at a time, with the mean number of mycotoxins per sample at 5.18 ± 2.26. Only 0.4% of samples did not contain mycotoxins. The most prevalent mycotoxin group in corn silage was the emerging mycotoxins found in 93.8% of samples, with type B trichothecenes also in a high percentage of samples at 89.5% (Table 4). Fumonisins were detected in 67.3% of samples. Total aflatoxins (AFs; aflatoxin B1 (AFB1), aflatoxin B2, aflatoxin G1, aflatoxin G2), type A trichothecenes, ZEA, *Penicillium* mycotoxins and ergot toxins were all detected at similar rates of 21.2%, 28.9%, 17.8%, 16.1% and 20.2%, respectively. The other mycotoxin groups were detected in less than 10% of samples.

All 35 measured mycotoxins were detected in corn silage samples (Table 5). Prevalence rates of individual mycotoxins followed a similar trend to corn grain, with the most prevalent mycotoxin being FA (93.8%) followed by DON (88.2%). The average concentration of FA in corn silage samples was 660 ± 805 µg/kg, with a median value of 361 µg/kg, a maximum of 5190 µg/kg and 75% of positive samples having concentrations less than 838 µg/kg. Deoxynivalenol was detected at a mean of 1870 ± 2440 µg/kg, median of 968 µg/kg and maximum of 16,603 µg/kg. The top 25% of samples had DON concentrations greater than 2189 µg/kg.

Other prevalent mycotoxins included the FBs, with FB1 present in 58.3%, FB2 in 47.4% and FB3 in 37.1% of corn silage samples. Of these three forms, FB1 was detected at the highest concentrations with a mean of 1990 ± 4350 µg/kg, median of 496 µg/kg, maximum of 45,900 µg/kg, and 75% quartile of 1549 µg/kg. The fungal metabolite of DON, 15ADON, appeared in 39.1% of samples with a mean concentration of 594 ± 684 µg/kg (median 342 µg/kg) and maximum of 3810 µg/kg. There was a greater prevalence of HT2 in corn silage (19.0%) than grain (5.9%). The mean concentration of HT2 in corn silage samples was 247 ± 547 µg/kg (median 66.4 µg/kg) and maximum detected concentration of 3710 µg/kg. Zearalenone was detected in a slightly lower percentage of corn silage (17.8%) samples than grain samples, but at a higher mean concentration of 560 ± 286 µg/kg, median of 320 µg/kg and maximum of 4020 µg/kg. The top 25% of samples had ZEA levels above 781 µg/kg.

*Penicillium* mycotoxins, comprising patulin, mycophenolic acid (MPA), roquefortine C and penicillic acid (PA), had a higher occurrence rate in corn silage than corn grain. Penicillic acid was the primary mycotoxin detected in this group with an occurrence rate of 10.9%. The mean concentration of PA was 192 ± 224 µg/kg, median of 123 µg/kg and maximum of 1410 µg/kg, but a 75% quartile of 205 µg/kg. Interestingly, AFB1 was detected in 7.9% of samples which was greater than corn grain with only 1.7% of samples containing AFB1. Although the concentrations for AFB1 were lower on average (mean 9.5 ± 13.0 µg/kg, median 4.6 µg/kg, 75% quartile of 11.5 µg/kg), there was a maximum of 82.4 µg/kg detected in corn silage.

### 2.2. Mycotoxins Exceeding Action, Advisory or Guidance Levels

Mycotoxin action, advisory or guideline limits are provided by the United States Food and Drug Administration (FDA) and European Commission (EC) for corn and corn-based products [8,9]. Although the listed limits may not be relevant for all animal species, investigation of the percentage of both corn grain and silage samples above each limit are provided to assess not only the mycotoxin content but also as a means for comparing the difference between these two feedstuffs (Table 6). Results of corn grain analysis showed that only 0.14% of corn grain samples had concentrations of AFB1 above 20 µg/kg although the same 0.14% also exceeded the FDA action level of 300 µg/kg for AFs. Concentrations of DON exceeded 5000 µg/kg in 1.55% of samples and 8000 µg/kg in 0.98% of samples. A total of 17.72% of samples had FBs concentrations that exceeded 5000 µg/kg, 9.56% of sample also contained FBs at or above 10,000 µg/kg, 3.38% exceeded 20,000 µg/kg but no samples exceeded 60,000 µg/kg. Only 0.42% of corn grain samples had ZEA levels exceeding the EC limit of 2000 µg/kg, while ochratoxin A (OTA) levels did not exceed the EC suggested regulatory limit.

Aflatoxin B1 concentrations in corn silage were detected above 20 µg/kg in 1.16% of samples, AFs were detected over 20 µg/kg in 6.09% of silages but exceeding 300 µg/kg in only 0.09% of samples (Table 6). There were 7.88% of samples that had DON over 5000 µg/kg, 3.40% exceeded 8000 µg/kg, and 1.88% of silage samples were over 10,000 µg/kg DON. The concentration of FBs in corn silage were lower than grain, with only 7.88% of samples over 5000 µg/kg, 3.94% over 10,000 µg/kg, 1.07% exceeding 20,000 µg/kg and no samples exceeding 60,000 µg/kg. The concentrations of ZEA in corn silage exceeded 2000 µg/kg in 1.25% of silages. Levels of OTA did not exceed the EC suggested regulatory limit.

### 2.3. Mycotoxin Co-Occurrence

#### 2.3.1. Frequency of Mycotoxin Pairs

**Figure 1:**
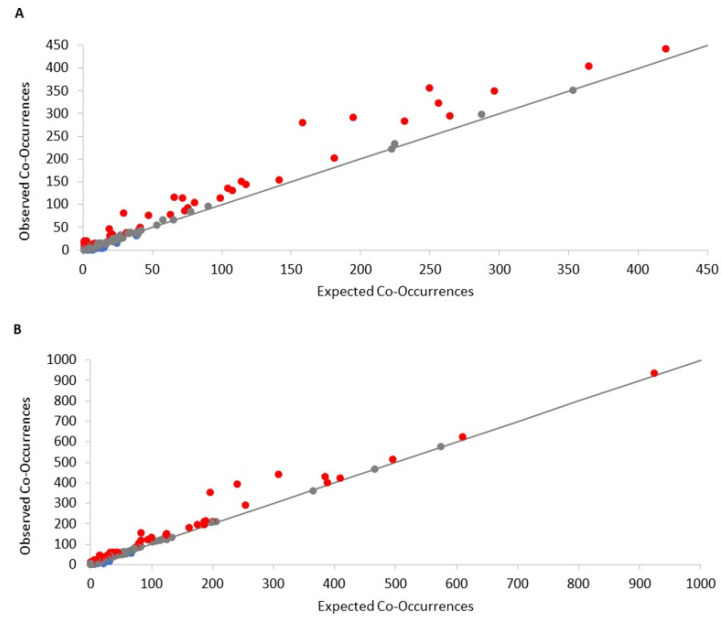
Plots showing the number of incidences of co-occurrence between selected pairs of individual mycotoxins for corn grain (**A**) and silage (**B**) samples. Red color depicts mycotoxin pairs with positive co-occurrences that were detected more than (*p* < 0.05) the model expected under a random association, blue depicts pairs with negative co-occurrences that were detected less than (*p* < 0.05) the model expected under a random association, and grey indicates those pairs that were equivalent to what the model expected under a random association.

Most frequencies of co-occurrence among mycotoxin pairs for corn grain were less than 100, although it ranged from 0 to over 400 (Figure 1A). Many of these mycotoxin pairs demonstrated a positive relationship, indicating that frequencies of co-occurrence were greater than an association by random chance (*p* < 0.05). Pairs of mycotoxins that co-occurred less than a random association, i.e., a negative relationship, generally had fewer frequencies of co-occurrence. There was a greater number of significant (*p* < 0.05) positively associated (56) pairs than negatively associated (23) pairs of mycotoxins. Additionally, there were 176 pairs of mycotoxins that had non-significant relationships, or a random association of co-occurrence.

The distribution of frequencies of co-occurrence among mycotoxin pairs in corn silage (Figure 1B) were similar to corn grain, although there were some pairs with much higher frequencies of co-occurrence at over 900 pairings. There was a greater number of significant (*p* < 0.05) pairs with a positive relationship (87) than pairs with a negative relationship (25). Similar to corn grain, many mycotoxin pairs (294) exhibited a random association in their frequency of occurrence.

#### 2.3.2. Probability of Mycotoxin Pairs

For corn grain samples analyzed, the most frequently detected co-occurrence was between DON and FA with a probability of 0.591 (*p* < 0.001; Table 7). Fusaric acid also had a relatively high probability of co-occurrence with FB1 (0.513), FB2 (0.417), FB3 (0.326), and 15ADON (0.372, *p* < 0.001). Similarly, there was a relatively high probability of co-occurrence between DON and 15ADON (0.361, *p* < 0.001). There were also relatively low, but significant, co-occurrences between DON and 3ADON (0.106, *p* < 0.001) and D3G (0.147, *p* < 0.001). Each of these mentioned pairs demonstrated a positive relationship and co-occurred at frequencies greater than what would be expected by a random association. Conversely, OTA co-occurred with DON (0.034, *p* = 0.028), 15ADON (0.021, *p* < 0.001), and D3G (0.009, *p* <0.001) at frequencies lower than a random association.

The results of corn silage analysis identified many similar pairs of mycotoxins that co-occurred more frequently than by chance (Table 8). The highest positive association was between DON and FA, with a probability of co-occurrence of 0.827 (*p* < 0.01). The occurrence of DON with 15ADON (0.345, *p* < 0.001) and FA with 15ADON (0.367, *p* = 0.001) were also relatively high in frequency of co-occurrence. Similar to corn grain, FA also had relatively high co-occurrence probabilities with FB1 (0.513, *p* < 0.001), FB2 (0.417, *p* < 0.001) and FB3 (0.326 *p* = 0.004). Furthermore, the mycotoxin pairs of OTA and HT2, OTA and FB2, 15ADON and MPA, and ZEA with PA, were all detected less frequently than by chance, which is characteristic of a negative relationship.

### 2.4. Mycotoxin Variation by Year

Significant (*p* < 0.01) yearly variations of mycotoxin concentrations in corn grain were observed for all groups except type A trichothecenes and *Penicillium* mycotoxins (Table 9). The highest mean concentration of type B trichothecenes occurred in 2019 (1673.4 ± 414.2 µg/kg), while FBs were greatest in 2018 with a mean of 7205.6 ± 593.3 µg/kg. Mean ZEA concentrations were above 200 µg/kg for all years except 2014 and 2015. Concentrations of AFs were below 5 µg/kg except for 2017 (310.1 ± 301.1 µg/kg) and 2019 (19.2 ± 2.9 µg/kg). The emerging mycotoxins, although one of the most frequent groups, remained at lower levels, at or below 471.7 ± 179.1 µg/kg for all years.

Concentrations of mycotoxins in corn silage were significantly different (*p* < 0.05) between years for all mycotoxin group types (Table 10). Mean concentrations of AFs were above 20 µg/kg in 2013, 2014, 2016 and 2019. Type B trichothecene means were above 1000 µg/kg for all years, with the maximum mean concentration observed in 2017 (2708.5 ± 266.3 µg/kg) and closely followed by 2018 (2353.7 ± 153.2 µg/kg). Type A trichothecenes were highest on average in 2017 at 660.3 ± 138.8 µg/kg, as was ZEA (2233.2 µg/kg). Emerging mycotoxins were observed in corn silage at concentrations higher than that of corn grain for all years, ranging between 378.3 ± 38.4 and 1778.4 ± 301.8 µg/kg. The *Penicillium* mycotoxins had the greatest mean concentrations in 2014 (214.0 ± 145.9 µg/kg) and 2017 (212.9 ± 68.9 µg/kg), while mean *Aspergillus* mycotoxin concentrations were greater in 2015 (722.9 µg/kg) and 2019 (237.6 µg/kg).

**Figure 2:**
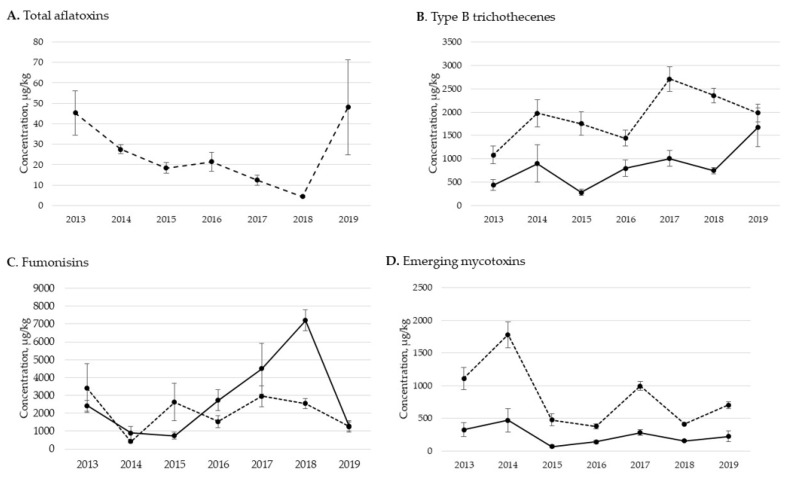
Yearly variation from 2013 to 2019 in concentrations (µg/kg) of total aflatoxins (**A**), type B trichothecenes (**B**), fumonisins (**C**) and emerging mycotoxins (**D**) in corn grain (solid line) and silage (dashed line). Total aflatoxins are the sum of aflatoxin B1 + aflatoxin B2 + aflatoxin G1 + aflatoxin G2, type B trichothecenes is the sum of deoxynivalenol + 3-acetyl-deoxynivalenol + 15-acetyl-deoxynvalenol + deoxynivalenol-3-glucoside + nivalenol + fusarenon X, fumonisins is the sum of fumonisin B1 + fumonisin B2 + fumonisin B3, and emerging mycotoxins the sum of fusaric acid + alternariol. Values show mean ± SE for each year. Each year represents new crop samples collected between September and December of each year. Only mycotoxins for which there were 10 or more positive results for each year per feedstuff are shown.

Mycotoxin groups detected in at least 10 samples for each year for each feedstuff were graphed (Figure 2). This criterion was met for AFs, type B trichothecenes, FBs and emerging mycotoxins. Only corn silage consistently had 10 or more positives for AFs in each year. The mean concentrations of AFs decreased from 2013 to 2018 but were again increased in 2019. Graphing the results for type B trichothecenes showed that the mean mycotoxin concentrations were numerically higher in corn silage than grain. Both feedstuffs had a general upward trend for the mean concentrations of type B trichothecenes, although there was variability between years. Emerging mycotoxins were also shown to have numerically higher mean levels in corn silage than grain. Conversely, FBs did not show a clear pattern for the concentrations in grain versus silage.

## 3. Discussion

Corn, as both a grain and forage source, is an important component of rations for livestock. However, this crop is susceptible to contamination with mycotoxins which can adversely impact the performance and health of animals. When naturally mycotoxin contaminated feedstuffs are consumed, unexpected health challenges can be observed including, but not limited to, gastrointestinal and internal organ damage, immune suppression, altered reproductive performance, lowered antioxidant status, reduced growth rates and poorer feed efficiency [10,11]. Mycotoxins do not need to be consumed at high levels to result in challenges to the animal. In fact, exposure to mycotoxins chronically and/or at lower concentrations that are below levels suggested by regulatory organizations, can negatively impact animal performance [7,12]. Furthermore, consumption of multiple mycotoxins simultaneously can further increase risk to the animal [12,13,14]. As a result, even minimal exposure to multiple mycotoxins could impact the efficiency and profitability of a farm.

Multiple mycotoxins were the norm in the samples analyzed, with around 5 mycotoxins per sample on average for both corn grain and corn silage and up to 12 or 13 mycotoxins present, respectively. Furthermore, 90.2% of grains and 96.5% of silages had at least two mycotoxins. Surveys completed in other countries investigating mycotoxin occurrence have also shown a presence of multiple mycotoxins in feedstuffs. In Belgium, whole plant corn silage analyzed over a three-year period had five or more mycotoxins in 46.7% of samples [5]. Reisinger et al. [15] reported an average of 13 mycotoxins in corn silage, with 87% of samples containing at least 5 mycotoxins out of the 61 mycotoxins analyzed.

Our present analysis reports the mycotoxin levels in both feedstuff types around harvest. It was clear that most mycotoxins detected at this timepoint were *Fusarium* mycotoxins, which made up the top 11 mycotoxins detected in grain and the top 9 mycotoxins detected in silage. The most prevalent mycotoxin was FA, one of the emerging mycotoxins, in 78.1% of corn grain and 93.8% of silage samples. Fusaric acid is an unusual mycotoxin in that it can be produced by at least 12 different *Fusarium* species [16]. As such, FA has been speculated to be one of the most widely produced mycotoxins. We confirmed this speculation in our survey, but FA may not always be the most frequent. Reisinger et al. [15] reported only 22% of 158 corn silage samples were positive for FA although more than 70% of samples contained other emerging mycotoxins such as beauvericin and enniatins. Although consumption of FA by animals can elevate brain serotonin levels, decrease blood pressure and act as a chelating agent that could be involved in abnormal bone formation, it does appear to have lower toxicity than other mycotoxins such as AF, DON or T2 [17,18,19].

The other two top mycotoxins detected in this survey were DON and FB1 which were present in 75.7 or 65.7% of grain and 88.2 or 58.3% of silage samples, respectively. In accordance with our results, DON and FBs are also shown to be frequently detected mycotoxins in European feeds for poultry with a prevalence of 98% and 100%, respectively [7]. Assessment of imported raw corn grain from the US to Korea also showed a high occurrence of DON and FB1, both in 100% of samples [20].

When investigating the frequency of these most prevalent *Fusarium* mycotoxins to exceed regulatory guidelines, 1.6% of corn grain had over 5000 µg/kg DON which is the FDA guidance for DON contaminated grains for swine [8]. This guidance states that corn and corn products exceeding 5000 µg/kg should be included at no more than 20% of the ration in order to limit total ration DON intake to 1000 µg/kg. Only 0.98% of grains had over 8000 µg/kg, the EC guidance level, with stipulations for complete rations not to exceed 900 µg/kg for pigs [9]. When considering corn silage, 3.4% of samples exceeded the EC limit of 8000 µg/kg while 1.9% exceeded the 10,000 µg/kg FDA advisory level for grains for poultry and ruminants, which should not exceed 50% in the final ration. Similarly, Birr et al. [21] reported that 9% of corn silage from Germany exceeded 5000 µg/kg. Despite fewer samples having very high DON levels, we did find that the mean concentration of DON in corn grain samples was 840 µg/kg. The presence of any level of DON, whether at lower or higher levels, could be problematic for animal health and performance. In fact, pigs consuming 900 µg/kg were shown to have reduced average daily gain as well as intestinal tract changes characterized by reduced villus height, increased crypt depth, and altered intestinal barrier function [22]. A meta-analysis by Holanda and Kim [23] showed that the consumption of 1000 µg/kg DON by pigs reduced average daily gain by 8.9% while House et al. [24] indicated that the same concentration of DON could significantly increase the number of days required for female pigs to reach 110 kg. Broilers consuming feed with DON at 2264 µg/kg have also shown reduced gain and increased feed conversion ratio [25]. In dairy cows, it is suggested that cows consuming dietary DON ≥ 6740 µg/kg can have 3.7 times greater abortion rates while dietary DON ≥ 3210 µg/kg may increase the percent of cows with endometritis by 1.9 times [26].

Fumonisin guidelines are provided by both FDA and EC. Our survey indicated a potential for FBs to exceed guidance levels, with 17.7% of samples containing FBs concentrations over the 5000 µg/kg FDA guidance level for corn designated for horses and rabbits. These grains should not exceed 20% of the diet [8]. Horses are particularly sensitive to FBs and may develop equine leukoencephalomalacia, a neurotoxic disease, if exposed [27]. We observed fewer samples that exceeded FBs levels suggested for other animal groups, with only 3.38% of grains exceeding the 20,000 µg/kg FDA guidance level for corn designated for swine and catfish and only 0.98%(grain) and 0.45% (silage) exceeded the 30,000 µg/kg guidance for corn for breeding ruminants and poultry. Although few samples exceeded these higher levels of FBs, research suggests that lower levels could impact animal health. The consumption of FBs as low as 5000 µg/kg are shown to increase *E. coli* colonization in the colon of pigs [28].

Our data also shows that ZEA could be a problematic mycotoxin in some cases as it contaminated about 20% of corn grain and silage. Guidance levels for ZEA are not currently provided by FDA, but 1.25% of silages contained over the EC guidance of 2000 µg/kg. Generally, ZEA consumption is not considered to impact growth performance, and in fact may even improve growth performance outcome, but ZEA does play a role in other areas of health [23]. For example, Wu et al. [29] showed that ZEA at 200 µg/kg can reduce serum immunoglobulins, increase inflammatory cytokines, and reduce concentrations of luteinizing hormone and estradiol in gilts. Additionally, dairy cows may be 1.8 times more likely to develop hyperketonemia when consuming dietary ZEA ≥ 90 µg/kg [26]. Although not considered for regulatory purposes, our data did show that 11.3% of grain and 12.6% of silage samples contained ZEA that exceeded 100 µg/kg. Depending on feedstuff inclusion rate in the ration, animal performance could be influenced by this presence of ZEA.

Fewer mycotoxins analyzed belonged to the *Aspergillus* or *Penicillium* groups. Aflatoxin B1, most frequently produced by *Aspergillus flavus*, is one of the most widely regulated mycotoxins globally [30]. This mycotoxin was detected in only 1.7% of grain and 7.9% of silage samples. The low occurrence rates may have been because a higher percentage of samples were received from Northern, rather than Southern, US locations. Aflatoxin is considered to be more prevalent in the Southern US, although it could occur in any region experiencing high temperatures and drought stress [3]. Furthermore, the distribution of AFs tends to be more heterogeneous in a feedstuff as opposed to a mycotoxin such as DON with a more uniform contamination pattern [31]. Despite AFB1 having a lower occurrence rate, it is still important to consider this mycotoxin for its impacts on animal health and associated human health through the direct consumption of contaminated plant or animal products such as milk. In fact, we did observe that 0.14% of grain and 1.2% of silage samples did contain over 20 µg/kg, the maximum level set by EC for grains and grain products [9]. The FDA action level for corn and corn products for immature animals, pets and dairy cattle is for AFs at 20 µg/kg, which we detected at over this level in 0.14% (grain) and 6.1% (silage) of samples [8]. As such, silage appears to be more likely to contain AFs but may not contain concentrations at any higher levels than grain.

Other mycotoxins in the *Penicillium* and *Aspergillus* groups can play an important role during storage of feedstuffs. These molds can grow at a wider range of pH, water activity and temperatures than *Fusarium* species, and thus are more abundant during storage [32]. Silages are higher moisture feedstuffs which increases the risk of mold growth. If silages are poorly fermented, have poor packing density or are not covered adequately, the risk of mycotoxin production can be further increased in addition to a decrease of the nutritional value of the poorly preserved and contaminated silage. In our survey, we did not observe high frequencies or high concentrations of these storage type mycotoxins. This observation is likely because we were focused on assessing new crop quality with fresh corn silage samples being collected and submitted for mycotoxin analysis at, or shortly after, harvest. As a result, these silage samples did not have an opportunity to be influenced by typical farm storage conditions. Although silage samples contained only about half a mycotoxin more than corn grain in our samples, it would be interesting to conduct further analysis and determine if the number of mycotoxins, or their concentrations, increase throughout storage.

Ergot alkaloids, produced by members of the genus *Claviceps,* are known to develop in small grains such as wheat, oats, and barley [33]. These toxins may also be present in grasses. Interestingly, the ergot alkaloid methylergonovine was observed in 12.7% of corn silage samples in our survey. Methylergonovine is a derivative of ergonovine and is used therapeutically for routine management of postpartum uterine atony and hemorrhage [33]. Although small grains are susceptible to ergot contamination, the only ergot producing fungus shown to develop in corn is *Claviceps gigantea*, which appears to be geographically limited to high altitudes of Mexico [34]. The ergot alkaloids we detected is more likely a result of cross contamination by other plants in the field such as the presence of small grains or weeds. In fact, Naude et al. [35] reported that after extensive investigation, the contamination of corn silage with ergot alkaloids was due to the presence of yellow nut sedge. Yellow nut sedge is a common weed found worldwide and is known to be frequently contaminated with *Claviceps cyperi* which can produce ergot alkaloids [35,36]. As such, silage samples analyzed in our survey may have contained weeds such as yellow nut sedge, which not only add to the mycotoxin content of a particular feedstuff but also increase the multiple mycotoxin risk of the whole ration.

Assessing combinations of mycotoxins may be even more important than detecting mycotoxins individually as there are many mycotoxins that may have additive, synergistic or even antagonistic interactions [17,37,38,39,40]. In both corn grain and corn silage, the most frequently encountered mycotoxin pair was DON and FA which occurred together in 59.1% (grain) and 82.7% (silage) of contaminated samples. Furthermore, our analysis demonstrated that the frequency of their co-occurrence was higher than a random association. Particularly for silage, due to the high probability of co-occurrence, it may be assumed that when there is a presence of one of these mycotoxins there will also be a presence of the other. The co-occurrence of DON and FA together in feedstuffs could be harmful for animal performance and health. These two mycotoxins are suspected to have synergistic relationships and when consumed simultaneously by pigs are shown to further depress weight gain compared to the mycotoxins individually [17]. A suspected mechanism of toxicological synergism between FA and DON relates to tryptophan metabolism. Tryptophan is primarily carried through the blood bound to albumin, but only free tryptophan can cross the blood–brain barrier. Fusaric acid is shown to compete with tryptophan for albumin binding sites resulting in increased unbound tryptophan [17]. As a result, there are increased levels of free tryptophan which is taken up by the brain and used to synthesize serotonin. Although DON and other trichothecenes do not alter the tryptophan concentration, they are shown to increase the serotonin turnover in the hypothalamus to result in DON-induced feed refusal and lethargic behavior [41]. As a result of these complementary actions, the interaction between DON and FA could cause animals to display greater symptoms of DON toxicity than would be expected based on the mycotoxin analysis of feedstuffs or rations.

We also observed that FA co-occurred at a high rate with FB1 in over 51% of corn grain (51.3%) and silage (54.7%) samples. Again, this prevalence of co-occurrence was found at a significantly greater rate than what would be expected by a random model. Since FA is uniquely produced by a number of different *Fusarium* species, particularly *F. moniliforme* which also is the primary producer of FB1, it is likely that FA will be present with other *Fusarium* mycotoxins [16,42]. Interestingly, there was no significant relationship of co-occurrence between DON and FB1 indicating a random association, although they did occur together at a higher rate in about 50% of samples. Despite the lack of a significant relationship, the presence of these two mycotoxins together could still impact animal performance and are shown to further the negative effects on internal organs, immunity and body weight when consumed together than alone [43,44].

When considering the risk from type B trichothecenes, DON should not be the only mycotoxin to consider. Our survey showed the co-occurrence of DON with other type B trichothecenes, including the two acetylated derivatives of DON, 3ADON and 15ADON. It is expected that these three toxins will appear together as they are produced by the same fungal biosynthetic pathway [45]. Other survey data has shown a strong correlation between DON and its derivatives [5]. Our results also showed that 15ADON was more prevalent than 3ADON, which is important when considering mycotoxicosis to an animal. In fact, 15ADON is shown to have higher toxicity than 3ADON, and higher or equal toxicity to DON, resulting in more histological lesions in the intestine both *ex vivo* and *in vivo* [38,46]. Furthermore, the co-contamination of DON with 15ADON, which in our survey co-occurred in 36.1% (grain) and 34.5% (silage) of samples, can further increase toxicity with these mycotoxins being shown to have additive and synergistic effects but could also have some antagonistic relationships depending on the mycotoxin concentrations [38]. Vandicke et al. [5,47] saw that the concentrations of DON and 15ADON have strongly positive correlation coefficients in both fresh (r = 0.70) and ensiled (r = 0.79) corn silage over a three-year period. As such, their research showed that the concentrations are linked, while our research showed that they have a reasonably high rate of co-occurrence. Although regulatory levels are based on DON only, the presence of DON derivatives and conjugates, such as 15ADON, should be considered when assessing total mycotoxin risk.

The strong positive probability of co-occurrence between many of the mycotoxin pairs may be an indication of similar environmental conditions required for molds to produce mycotoxins, the prevalence of the different mold types or the types of mycotoxins a mold can produce. As mentioned previously, FA can be produced by a variety of different mold types [16]. As such, FA may be produced under a variety of climatic conditions leading to its high co-occurrence with other mycotoxins. Conversely, the production of DON and FBs are promoted by contrasting environmental conditions [48]. These two mycotoxins may have a high rate of co-occurrence, which we observed in both corn grain and silage, but there was a random association with no significant relationship between the two. Furthermore, the contamination of OTA with *Fusarium* mycotoxins was most likely to result in co-occurrence that was observed to a lesser extent than what would be expected randomly. Interestingly, OTA production in cereals is assumed to be from *Penicillium verrucosum* in temperate regions such as Europe and Canada [48]. Infection of this mold and its production of OTA is thought to occur only post-harvest during the drying stage when there is slow drying in conjunction with rain or fog. This somewhat different contamination pattern of OTA versus other mycotoxins could lead to the observed lower co-occurrence rates.

Yearly variations in occurrence and concentration of mycotoxin groups were observed. Environmental conditions during plant growth and harvest play a significant role in year-to-year variation of mycotoxin type and content. Furthermore, different weather conditions may lead to varying mycotoxin profiles. For example, the production of AFs and FBs are associated with preharvest drought stress while DON is produced during periods of excess moisture at anthesis [48]. As such, it was anticipated that there could be yearly variations because of fluctuating weather patterns.

Based on our data, we observed a general upward trend in the concentrations of type B trichothecenes, with 2017, 2018 and 2019 containing the higher mean levels of this mycotoxin group. Historical data reported by the National Oceanic and Atmospheric Administration (NOAA) indicate an upward trend in precipitation levels in the contiguous U.S., with 2019 being the second wettest year on record since 1973 [49]. Mueller at al. [50] reported that weather in the United States in 2018 was characterized by unfavorably wet conditions and delayed harvest which resulted in an estimated 2.5 billion bushels of corn grain contaminated with mycotoxins. Our survey showed frequent mycotoxin contamination of grain and silage in 2018, although 2018 was not necessarily the year with highest mean levels of contamination. Interestingly, AFs in corn silage were also shown to have a higher mean level in 2019. Typically, AFs are shown to develop during dryer conditions. This response may have been a result of localized drought conditions despite the general U.S. having higher precipitation, or this increase in AFs may have occurred at or after harvest rather than in the field. It should be noted that there were higher numbers of samples of grain and silage collected in 2016 through 2019 than in 2013–2015. This difference in sample number could have played a role in the detection of mycotoxins.

Regarding the difference between corn grain and silage, it was observed that silage typically had greater yearly concentrations of mycotoxins than grain. Research in wheat showed DON contamination levels to be greatest in glumes and straw, and significantly lower in the grain [51]. In corn, *Fusarium* mycotoxins are reported to contaminate leaves and stalks more than kernels [52]. Investigation of *Fusarium* colonization on ears and stalks of corn showed that some species tend to grow on the grain while others are more inclined to grow on stalks, whereas others such as *F. graminearum* are likely to develop on both ears and stalks but are highly dependent on plant growth stage and environmental conditions [53]. It is reasonable to assume that silage could have higher mold and mycotoxin levels due to the nature of including both stems and leaves which not only increases surface area exposed but may also bring in additional contamination sources such as mold contaminated soil. An exception for this tendency for higher mycotoxins in silage was with FBs, where corn grain and silage did not show a clear pattern of one containing higher concentrations than the other which may have been due to a variety of factors including the timing of weather events promoting FBs development.

## 4. Conclusions

Results of our survey represent a snapshot of the mycotoxin contamination of corn grain and corn silage at harvest from across the United States between 2013 and 2019. A total of 711 corn grain and 1117 corn silage samples were analyzed for the presence of 35 mycotoxins. Multiple mycotoxin contamination was the norm, with a mean of about five mycotoxins per sample. The majority of mycotoxins detected in this survey are considered to be produced during field growth of the crop, while the presence of storage-type mycotoxins was minimal. However, certain mycotoxins are known to increase during storage and a next step would be for future surveys to track mycotoxins throughout storage. Mycotoxin concentrations did vary by year, as expected due to the role that environmental conditions can play on mold growth and mycotoxin development.

*Fusarium* mycotoxins were prevalent, with FA, DON, FBs and 15ADON being the most frequently observed mycotoxins in both grain and silage. These mycotoxins had high rates of co-occurrence. The most frequent pair was DON and FA in 59% of grain and 83% of silage samples. The presence of FB1 with FA was just over 50%, while there was also a relatively high co-occurrence between DON and its metabolite 15ADON. Each of these interactions resulted in co-occurrences that occurred more than what would be expected by random association. The presence of multiple mycotoxins could lead to an increase in detrimental effects to the animal upon consumption of contaminated feed materials. Furthermore, some samples contained concentrations of mycotoxins above guidelines indicated by governing agencies, particularly DON and FBs. Considering the presence of higher concentrations of mycotoxins in both grain and silage, the frequent co-occurrence of several important *Fusarium* mycotoxins, as well as the yearly variability of mycotoxins, the assessment and quantification of multiple mycotoxins in both corn grain and silage should be considered routine when developing a mycotoxin control program.

## 5. Materials and Methods

### 5.1. Sample Collection

A total of 711 corn grain and 1117 corn silage samples that represented 7 harvests from 2013 to 2019 (samples collected between September and December of each year) were collected from across the United States (Table 11). Samples were submitted on a voluntary basis from animal production farms and animal feed production facilities to the Alltech Annual Harvest Analysis Survey. This survey focuses on assessing mycotoxin risk in new crop samples each year. Instructions for proper sampling procedures based on methods described by the USDA Grain Inspection Handbook [54] and Undersander et al. [55] were available to individuals collecting samples. These procedures outline the process of collecting subsamples and creating a final homogenous sample. Submission instructions specified that each sample be packaged to preserve the integrity of mycotoxin contents (i.e., double bagged, vacuum sealed, and refrigerated at <4 °C). It was recommended that samples be immediately submitted to the laboratory after collection and sent via overnight shipping. A final weight of 200–400 g was requested for samples sent to the laboratory for mycotoxin analysis.

Corn sample locations represented 26 states, with the majority coming from the Midwestern United States (50.4%), followed by the Northeast (28.3%) and Southeast (17.6%). Only a small proportion of corn samples submitted for analysis were sourced from the Southwest (2.0%) or West (1.8%). Samples of corn silage were sourced from 36 states and primarily submitted from the Midwest (48.3%), Northeast (25.6%) or West (18.2%). Fewer samples were received from the Southeast (6.2%) or Southwest (1.8%).

### 5.2. Mycotoxin Quantification

A total of 35 individual mycotoxins were analyzed for each corn grain or corn silage sample submitted (Table 12). These mycotoxins were grouped into 10 groups: total aflatoxins (AFB1 + aflatoxin B2 + aflatoxin G1 + aflatoxin G2), ochratoxins (OTA + ochratoxin B), type B trichothecenes (DON + 3ADON + 15ADON + D3G + nivalenol + fusarenon X), type A trichothecenes (T2 + HT2 + diacetoxyscirpenol + neosolaniol), fumonisins (FB1 + FB2 + FB3), ZEA, emerging mycotoxins (FA + alternariol), *Penicillium* mycotoxins (patulin + MA + roquefortine C + PA), *Aspergillus* mycotoxins (wortmannin + gliotoxin + sterigmatocystin + verruculogen), ergot alkaloids (ergometrine + ergotamine + ergocryptine + lysergol + methylergonovine).

Analysis for the simultaneous presence of the 35 mycotoxins was carried out by the Alltech 37+^®^ Analytical Services Laboratory located in Nicholasville, KY, USA (ISO/IEC 17025:2005 No. 79481, Certificate No. L14-281 and ISO/IEC 17025:2017 official accreditation No. 79481, Certificate No. L20-392, Perry Johnson Laboratory Accreditation, Inc.) following methods published by Jackson et al. [56]. Samples were processed immediately upon receipt. Silage samples were freeze dried for ≥36 h to remove water whereas the moisture content of corn grain was determined immediately. Each sample was then finely ground using an EK43 grinder (Mahlkönig GmbH and Co., Hamburg, Germany). Aliquots (400 mg ± 2%) of samples were placed in reaction tubes and spiked with 20 µL of a 1:1:1 internal standard mixture ([^13^C_15_]-DON at 4.5 µg/g; [^13^C_18_]-ZEA at 4.5 µg/g; [^13^C_17_]-AFB1 at 273 ng/g). Corn grain samples were extracted with 1600 µL and corn silage with 3200 µL of a mixture of acetonitrile/water/formic acid (84.0:15.9:0.1, *v*/*v*/*v*) for 18 h at room temperature (RT) with shaking (300 rpm, New Brunswick Scientific, Enfield, CT, USA). All samples were centrifuged at 12,000 rpm for 15 min (Beckman Coulter Inc., Fullerton, CA, USA). Supernatant (400 µL for corn grain; 800 µL for corn silage) was collected and added to an autosampler vial and dried under a N_2_ stream for 30 min at RT. Samples were reconstituted in 400 µL of loading buffer (LB) consisting of water/acetonitrile/formic acid (95.0:4.9:0.1, *v*/*v*/*v*) containing 10 mmol L^−1^ ammonium acetate. An internal standard solution (20 µL) of [^13^C_34_]-FB1 at 2.3 µg/g was added to each reconstituted sample before vials were placed in the UPLC autosampler. Salinized glass vials with a dichlorodimethylsilane solution were used throughout the study to prevent mycotoxin interaction with glassware.

Calibration standards were prepared from a stock solution containing a mixture of 35 mycotoxins. The solution was diluted 1:4 (*v*/*v*) with acetonitrile five times to yield the following dilutions 1(A):4(B):16(C):64(D):256(E):1024(F). The linear calibration curve spanned concentrations differing by 2–5 orders of magnitude, depending on the analyte. Blank samples and a certified reference material were used after the calibration curve was built, and a system suitability check using standard solution C was performed after every five injections. The recovered levels of certified mycotoxins were accurate within ± 25%.

Ultra-pressure liquid chromatography with tandem mass spectrometry (UPLC-MS/MS) analysis was performed on a Waters Acquity UPLC-TQD system (Waters Corp., Milford, MA, USA) following methods of Jackson et al. [56] and utilizing an ethylene-bridged hybrid (BEH) C18 analytical column (1.7 µm particle size, 2.1 × 100 mm, Waters Corp., Milford, MA, USA) maintained at 40 °C. Analyses were performed at 0.41 mL/min over 16 min per sample injection with a gradient of water (eluent A) and methanol (eluent B), both containing 0.1% (*v*/*v*) formic acid. The column was held at its initial condition (5% B) for 2 min, followed by a linear increase to 10% B over 2 min, followed by a linear increase to 75% B over 8 min, then maintained at 99% B for 2 min, and finally re-equilibrated for 2 min with 5% B. Full loop injections of 10 µL were performed with 4× loop overfill without further sample preparation.

The MS was operated under the following conditions: source temperature (150 °C), cone gas flow rate (20 L/h), desolvation gas flow rate (450 L/h), cone voltage (20 V), and capillary voltage (1 kV). Nitrogen (purity 99.0%; Nitroflow, Parker-Balston, Haverhill, MA, USA) was used as the desolvation gas and the cone gas. The MS was operated in both negative or positive ion multiple reaction monitoring (MRM) mode for each compound in 1 min segments centered on the retention time of the target analyte, based on [M+H]^+^, [M+NH_4_]^+^, [M-H]^-^ [M+formate]^-^ precursor ion formation. Peak width was estimated at 6 s and a desired value of 16 points per peak was chosen. The instrument was set to automatic dwell on each transition.

### 5.3. Statistical Analysis

We analyzed mycotoxin concentrations for each of the 10 groups of mycotoxins (described above) for corn and corn silage samples by year using analysis of variance (ANOVA). Mycotoxin concentrations were log transformed to satisfy assumptions of normality. Statistical analyses were performed with R using built-in functions [57].

We characterized the co-occurrence of mycotoxins present among samples by constructing a series of probabilistic models. These models examined pairwise co-occurrences of individual mycotoxins following the methods of Veech [58] and Griffith et al. [59]. Measures of co-occurrence were evaluated by the observed number of instances samples contained two individual mycotoxins relative to the expected number of times that these would occur together by random chance. Separate analyses were conducted for corn and corn silage samples and among individual mycotoxins (i.e., two analyses).

Matrices for each analysis were constructed comprising of 0′s and 1′s. Among samples, laboratory analyses that detected a mycotoxin, reported as a positive, non-zero concentration, were assigned a “1”, and analyses that did not detect a mycotoxin, reported as a zero, were assigned a “0”. We calculated the probability of co-occurrence *sensu* Griffith et al. [59] that a corn or corn silage sample would contain an individual mycotoxin given that it contained a second individual mycotoxin as,

(1) $$Pj=\frac{\left(\begin{array}{c} N_{x}\\ j \end{array}\right)\times \left(\begin{array}{c} N-N_{x}\\ N_{y}-j \end{array}\right)}{\left(\begin{array}{c} N\\ N_{y} \end{array}\right)}\;,$$

where *j* = 1 to *Nx* samples, *Nx* is the number of samples where mycotoxin *x* occurs, *Ny* is the number of samples where mycotoxin *y* occurs, and *N* is the total number of samples that were analyzed where both *x* and *y* mycotoxins could occur. The term $\left(\begin{array}{c} N_{x}\\ j \end{array}\right)$, is the number of ways of selecting *j* samples that have mycotoxin *x* given there are *Nx* samples that were analyzed. The term $\left(\begin{array}{c} N-N_{x}\\ N_{y}-j \end{array}\right)$, is the number of ways of selecting *Ny*–*j* samples that have mycotoxin *y* but not mycotoxin *x* given there are *N*–*Nx* sites. The term $\left(\begin{array}{c} N\\ N_{y} \end{array}\right)$, is the total number of combinations that the number of samples *Ny* could be obtained out of all *N* samples. A mycotoxin co-occurrence matrix was constructed along with an observed–expected plot to visually evaluate whether probabilities of observed frequencies of co-occurrence were greater than (red), less than (blue), or equivalent (i.e., random) to (grey) expected co-occurrences. This approach to analyzing species co-occurrence was performed in R with the package “cooccur” [59].

## Figures and Tables

**Table 1:** Distribution of the number of mycotoxins detected and their proportion in all samples of corn grain and silage.

	Corn Grain		Corn Silage	
Number of Mycotoxins	Number of Samples	Proportion (%)	Number of Samples	Proportion (%)
0	10	1.4	5	0.4
1	60	8.4	31	2.8
2–3	161	22.6	233	20.9
4–6	306	43.0	547	49.0
7–9	150	21.1	263	23.5
10–12	24	3.4	36	3.2
13 or more	0	0.0	2	0.2
Mean ± SD	4.79 ± 2.44		5.18 ± 2.26	
Minimum	0		0	
Maximum	12		13	

**Table 2:** Occurrence, mean (SD), median, 25% and 75% quartiles, and maximum concentrations (µg/kg) of mycotoxin positive samples by mycotoxin groups in corn grain (*n* = 711).

	Samples		Concentrations (µg/kg)				
Mycotoxin Groups ^1^	Number	%	Mean ± SD	Median	25% Quartile	75% Quartile	Maximum
Aflatoxin B1	12	1.7	58.1 (173)	7.1	4.5	14.6	606
Aflatoxins, total	54	7.6	15.2 (82.8)	2.7	2.0	4.6	611
Ochratoxins	48	6.8	4.9 (2.4)	5.5	2.2	6.4	11.6
Type B trichothecenes	581	81.7	900 (2020)	332	117	965	33,230
Type A trichothecenes	94	13.2	40.6 (71.8)	19.7	7.2	40.5	618
Fumonisins	493	69.3	4446 (7360)	1361	333	5333	59,117
Zearalenone	151	21.2	301 (486)	115	43.8	337	2894
Emerging mycotoxins	555	78.1	207 (460)	89.3	31.4	205	6792
*Penicillium* mycotoxins	65	9.1	166 (341)	13.6	5.2	134	1688
*Aspergillus* mycotoxins	44	6.2	50.9 (143)	4.2	3.8	9.5	608
Ergot alkaloids	23	3.2	4.0 (2.6)	3.5	1.7	5.5	10.2

^1^ Aflatoxins, total: aflatoxin B1 + aflatoxin B2 + aflatoxin G1 + aflatoxin G2; ochratoxins: ochratoxin A + ochratoxin B; type B trichothecenes: deoxynivalenol + 3-acetyl-deoxynivalenol + 15-acetyl-deoxynvalenol + deoxynivalenol-3-glucoside + nivalenol + fusarenon X; type A trichothecenes: T-2 toxin + HT-2 toxin + diacetoxyscirpenol + neosolaniol; fumonisins: fumonisin B1 + fumonisin B2 + fumonisin B3; emerging mycotoxins: fusaric acid + alternariol; *Penicillium* mycotoxins: patulin + mycophenolic acid + roquefortine C + penicillic acid; *Aspergillus* mycotoxins: wortmannin + gliotoxin + sterigmatocystin + verruculogen; ergot alkaloids: ergometrine + ergotamine + ergocryptine + lysergol + methylergonovine.

**Table 3:** Occurrence, mean (SD), median, 25% and 75% quartiles, and maximum concentrations (µg/kg) of positive samples for individual mycotoxins in corn grain (*n* = 711).

	Samples		Concentrations (µg/kg)				
Mycotoxins	Number	%	Mean (SD)	Median	25% Quartile	75% Quartile	Maximum
Aflatoxin B1	12	1.7	58.1 (173)	7.1	4.5	14.6	606
Aflatoxin B2	16	2.3	3.6 (1.2)	3.2	2.7	4.5	5.9
Aflatoxin G1	3	0.4	2.9 (3.1)	1.3	1.1	3.8	6.4
Aflatoxin G2	28	3.9	2.0 (0.5)	2.0	1.7	2.2	3.1
Ochratoxin A	32	4.5	5.5 (1.5)	6.0	5.3	6.5	7.0
Ochratoxin B	19	2.7	3.1 (2.0)	2.2	2.0	3.3	9.7
Deoxynivalenol	538	75.7	840 (1790)	333	129.2	914	26,974
3ADON ^1^	100	14.1	32.1 (50.3)	17.1	10.7	30.3	408
15ADON ^1^	339	47.7	91.6 (137)	52.1	25.2	96.5	1172
D3G ^1^	138	19.4	218 (454)	124	72.0	213	5084
Nivalenol	0	0	-	-	-	-	-
Fusarenon X	51	7.2	130 (477)	23.4	17.1	43.0	3204
T-2 toxin	53	7.5	18.7 (32.0)	9.6	6.6	17.5	227
HT-2 toxin	42	5.9	58.3 (63.9)	37.9	19.9	81.6	364
Diacetoxyscirpenol	13	1.8	10.7 (6.4)	7.6	6.1	15.4	24.1
Neosolaniol	15	2.1	15.4 (12.4)	13.8	5.7	23.9	45.5
Fumonisin B1	467	65.7	3800 (6100)	1340	390	4750	52,503
Fumonisin B2	380	53.4	738 (1150)	204	62.3	772	5643
Fumonisin B3	297	41.8	462 (651)	162	46.4	554	2802
Zearalenone	151	21.2	302 (486)	115	43.8	337	2894
Fusaric acid	555	78.1	207 (460)	88.7	31.4	205	6792
Alternariol	2	0.3	69.4 (31.1)	69.4	58.4	80.4	91.4
Patulin	2	0.3	106 (41.9)	106	91.6	121	136.0
Mycophenolic acid	44	6.2	202 (391)	17.1	5.5	190	1688
Roquefortine C	16	2.3	4.6 (2.8)	5.1	2.4	5.5	11.6
Penicillic acid	6	0.8	195 (193)	127	43.1	315	493
Wortmannin	3	0.4	150 (157)	129	66.7	222	315
Gliotoxin	6	0.8	340 (245)	423	124	498	608
Sterigmatocystin	33	4.6	4.0 (1.0)	3.8	3.5	5.1	5.3
Verruculogen	15	2.1	4.5 (0.8)	4.7	4.4	4.9	5.9
Ergometrine	1	0.1	3.8 (0)	3.8	3.8	3.8	3.8
Ergotamine	12	1.7	4.2 (2.3)	3.5	2.6	5.2	10.2
Ergocryptine	0	0	-	-	-	-	-
Lysergol	3	0.4	4.1 (3.5)	2.7	2.1	5.4	8.1
Methylergonovine	11	1.5	2.3 (1.4)	1.7	1.5	3.1	4.6

^1^ 3ADON: 3-acetyl-deoxynivalenol; 15ADON: 15-acetyl-deoxynvalenol; D3G: deoxynivalenol-3-glucoside.

**Table 4:** Occurrence, mean (SD), median, 25% and 75% quartiles, and maximum concentrations (µg/kg) of mycotoxin positive samples by mycotoxin groups in corn silage (*n* = 1117).

	Samples		Concentrations (µg/kg)				
Mycotoxin Groups ^1^	Number	%	Mean ± SD	Median	25% Quartile	75% Quartile	Maximum
Aflatoxin B1	88	7.9	9.5 (13.0)	4.6	2.2	11.5	82.4
Aflatoxins, total	237	21.2	18.0 (37)	7.1	2.9	22.8	423
Ochratoxins	24	2.1	9.7 (15.9)	4.9	2.2	9.5	76.3
Type B trichothecenes	1000	89.5	2169 (2900)	1090	456	2650	19,849
Type A trichothecenes	323	28.9	181 (459)	40.8	17.6	130	3712
Fumonisins	752	67.3	2227 (5100)	449	152	1665	53,046
Zearalenone	199	17.8	560 (683)	304	77.4	781	4021
Emerging mycotoxins	1048	93.8	661 (805)	361	152	840	5195
*Penicillium* mycotoxins	180	16.1	151 (216)	88.9	19.6	183	1411
*Aspergillus* mycotoxins	65	5.8	128 (241)	37.2	3.4	135	1232
Ergot alkaloids	226	20.2	16.5 (64.4)	3.2	1.7	9.0	811

^1^ Aflatoxins, total: aflatoxin B1 + aflatoxin B2 + aflatoxin G1 + aflatoxin G2; ochratoxins: ochratoxin A + ochratoxin B; type B trichothecenes: deoxynivalenol + 3-acetyl-deoxynivalenol + 15-acetyl-deoxynvalenol + deoxynivalenol-3-glucoside + nivalenol + fusarenon X; type A trichothecenes: T-2 toxin + HT-2 toxin + diacetoxyscirpenol + neosolaniol; fumonisins: fumonisin B1 + fumonisin B2 + fumonisin B3; emerging mycotoxins: fusaric acid + alternariol; *Penicillium* mycotoxins: patulin + mycophenolic acid + roquefortine C + penicillic acid; *Aspergillus* mycotoxins: wortmannin + gliotoxin + sterigmatocystin + verruculogen; ergot alkaloids: ergometrine + ergotamine + ergocryptine + lysergol + methylergonovine.

**Table 5:** Occurrence, mean (SD), median, 25% and 75% quartiles, and maximum concentrations (µg/kg) of positive samples for individual mycotoxins in corn silage (*n* = 1117).

	Samples		Concentrations (µg/kg)				
Mycotoxins	Number	%	Mean (SD)	Median	25% Quartile	75% Quartile	Maximum
Aflatoxin B1	88	7.9	9.5 (13.0)	4.6	2.2	11.5	82.4
Aflatoxin B2	70	6.3	15.6 (19.9)	6.1	3.0	22.8	97.1
Aflatoxin G1	39	3.5	3.1 (2.8)	1.8	1.1	4.9	9.8
Aflatoxin G2	66	5.9	33.5 (61.5)	21.0	11.6	31.2	423
Ochratoxin A	18	1.6	6.5 (8.1)	4.2	2.2	7.2	36.7
Ochratoxin B	6	0.5	19.3 (28.1)	9.6	6.5	11.6	76.3
Deoxynivalenol	985	88.2	1870 (2440)	968	413	2189	16,603
3ADON ^1^	214	19.2	116 (223)	52.4	24.8	114	2109
15ADON ^1^	437	39.1	594 (684)	342	177	714	3813
D3G ^1^	84	7.5	211 (313)	100	54.1	246	2202
Nivalenol	7	0.6	388 (338)	239	203	374	1130
Fusarenon X	40	3.6	635 (644)	408	188	920	2997
T-2 toxin	31	2.8	11.7 (19.6)	6.83	3.7	11.1	112
HT-2 toxin	212	19.0	247 (547)	66.4	30.3	195	3712
Diacetoxyscirpenol	50	4.5	41.8 (75.1)	12.1	9.8	29.2	446
Neosolaniol	74	6.6	48.0 (144)	11.6	7.7	23.3	912
Fumonisin B1	651	58.3	1990 (4350)	496	200	1549	45,922
Fumonisin B2	529	47.4	417 (863)	90.6	25.3	325	6875
Fumonisin B3	414	37.1	376 (630)	107	42.1	363	3597
Zearalenone	199	17.8	560 (286)	320	77.4	781	4021
Fusaric acid	1048	93.8	660 (805)	361	151	838	5194
Alternariol	6	0.5	47.4 (37.7)	34.6	25.2	71.1	104
Patulin	7	0.6	422 (286)	320	223	525	972
Mycophenolic acid	38	3.4	16.8 (27.8)	6.8	4.3	14.0	143.6
Roquefortine C	20	1.8	9.2 (21.0)	2.1	1.9	6.1	90.8
Penicillic acid	122	10.9	192 (224)	123	65.7	205	1410
Wortmannin	4	0.4	11.9 (5.5)	11.2	8.0	15.2	18.6
Gliotoxin	23	2.1	278 (329)	188	78.5	312	1231
Sterigmatocystin	24	2.1	3.5 (2.6)	2.9	1.9	4.4	13.2
Verruculogen	23	2.1	80.5 (149)	36.1	5.1	75.9	726
Ergometrine	22	2.2	5.7 (7.6)	3.1	2.5	3.9	34.2
Ergotamine	34	3.0	41.0 (72.6)	13.3	9.9	34.8	356
Ergocryptine	6	0.5	224 (297)	130	61.3	195	811
Lysergol	52	4.7	8.8 (13.1)	4.9	2.5	8.3	68.5
Methylergonovine	142	12.7	3.0 (5.2)	1.8	1.1	3.0	54.9

^1^ 3ADON: 3-acetyl-deoxynivalenol; 15ADON: 15-acetyl-deoxynvalenol; D3G: deoxynivalenol-3-glucoside.

**Table 6:** Percentage of analyzed samples with mycotoxin concentrations at or exceeding the United States Food and Drug Administration (FDA) and European Commission (EC) action, advisory or guidance levels for corn, corn products or complete feedstuffs intended for use in animal rations [8,9].

	Mycotoxin Limits, µg/kg ^1^					
	AFB1	AFs	DON	FBs ^2^	ZEA	OTA
Limit	20 *	20 **	5000 **	5000 **	2000 *	250 *
% Grain	0.14	0.14	1.55	17.72	0.42	-
% Silage	1.16	6.09	7.88	7.88	1.25	-
Limit		100 **	8000 *	10,000 **		
% Grain		0.14	0.98	9.56		
% Silage		0.27	3.4	3.94		
Limit		200 **	10,000 **	20,000 **		
% Grain		0.14	0.56	3.38		
% Silage		0.18	1.88	1.07		
Limit		300 **		30,000 **		
% Grain		0.14		0.98		
% Silage		0.09		0.45		
Limit				60,000 *^,^**		
% Grain				-		
% Silage				-		

^1^ AFB1: aflatoxin B1; AFs: aflatoxin B1 + aflatoxin B2 + aflatoxin G1 + aflatoxin G2; DON: deoxynivalenol; FBs: fumonisin B1 + fumonisin B2 + fumonisin B3; ZEA: zearalenone; OTA: ochratoxin A. ^2^ Fumonisins guidance by FDA is based on the sum of fumonisins B1 + B2 + B3, while EC guidance refers to the sum of fumonisins B1 and B2. * EC maximum and guidance levels for cereal and cereal products; ** FDA action, advisory or guidance levels for corn and corn products with different mycotoxin levels corresponding to intended use for different species or age groups.

**Table 7:** Probability of co-occurrence between pairs of 15 individual mycotoxins for corn grain samples. Numbers indicate probability of co-occurrence, bolded values indicate significant (*p* < 0.05) co-occurrence of groups that are detected more than (red), less than (blue) or equivalent to (grey) what the model expected under a random association. Pairs that did not co-occur among samples are blank (white) or not included in the table.

Mycotoxin ^1^	OTA	DON	3ADON	15ADON	D3G	T2	HT2	FB1	FB2	FB3	ZEA	FA	MPA	PA
AFB1		0.013	0.002	0.008	0.003			**0.011**	**0.09**	**0.007**	0.004	**0.013**		
OTA		**0.034**	0.006	**0.021**	**0.009**	**0.003**	0.003	**0.030**	0.024	0.019	0.010	0.035	0.003	
DON			**0.106**	**0.361**	**0.147**	0.056	**0.045**	0.497	0.404	0.316	**0.161**	**0.591**	0.047	0.006
3ADON				**0.067**	**0.027**	0.010	0.008	0.092	0.075	0.059	**0.030**	0.110	0.009	
15ADON					**0.093**	0.036	**0.028**	0.313	**0.255**	**0.199**	**0.101**	**0.372**	**0.030**	0.004
D3G						0.014	**0.011**	0.127	**0.104**	0.081	**0.041**	**0.152**	0.012	0.002
T2							**0.004**	0.049	0.040	0.031	0.016	**0.058**	0.005	
HT2								0.039	0.032	0.025	**0.013**	0.046	0.004	
FB1									**0.351**	**0.274**	**0.139**	**0.513**	0.041	0.006
FB2										**0.223**	**0.114**	**0.417**	0.033	0.005
FB3											**0.089**	**0.326**	0.026	0.004
ZEA												**0.166**	0.013	0.002
FA													0.048	0.007

^1^ AFB1: aflatoxin B1; OTA: ochratoxin A; DON: deoxynivalenol; 3ADON: 3-acetyl-deoxynivalenol; 15ADON: 15-acetyl-deoxynvalenol; D3G: deoxynivalenol-3-glucoside; T2: T-2 toxin; HT2: HT-2 toxin; FB1: fumonisin B1; FB2: fumonisin B2; FB3: fumonisin B3; ZEA: zearalenone; FA: fusaric acid; MPA: mycophenolic acid; PA: penicillic acid.

**Table 8:** Probability of co-occurrence between pairs of 15 individual mycotoxins for corn silage samples. Numbers indicate probability of co-occurrence, bolded values indicate significant (*p* < 0.05) co-occurrence of groups that are detected more than (red), less than (blue) or equivalent to (grey) what the model expected under a random association. Pairs that did not co-occur among samples are blank (white) or not included in the table.

Mycotoxin ^1^	OTA	DON	3ADON	15ADON	D3G	T2	HT2	FB1	FB2	FB3	ZEA	FA	MPA	PA
AFB1	0.001	0.069	0.015	0.031	0.006	0.002	0.015	0.046	0.037	0.029	0.014	0.074	0.003	**0.009**
OTA		0.014	0.003	0.006	0.001		**0.003**	0.009	**0.008**	0.006	**0.003**	0.015		0.002
DON			**0.169**	**0.345**	**0.066**	0.024	**0.167**	0.514	0.418	0.327	**0.157**	**0.827**	0.03	0.096
3ADON				**0.075**	**0.014**	0.005	0.036	**0.112**	**0.091**	**0.071**	**0.034**	**0.180**	0.007	0.021
15ADON					**0.029**	0.011	**0.074**	**0.228**	0.185	**0.145**	0.070	**0.367**	**0.013**	0.043
D3G						0.002	**0.014**	0.044	0.036	0.028	**0.013**	**0.071**	0.003	0.008
T2							**0.005**	0.016	0.013	0.010	0.005	0.026	0.001	0.003
HT2								**0.111**	**0.090**	**0.070**	**0.034**	0.178	0.006	0.021
FB1									**0.276**	**0.216**	0.104	**0.547**	**0.020**	0.064
FB2										**0.176**	**0.084**	**0.444**	**0.016**	0.052
FB3											0.066	**0.348**	0.013	**0.040**
ZEA												**0.167**	0.006	**0.019**
FA													0.032	0.102
MPA														0.004

^1^ AFB1: aflatoxin B1; OTA: ochratoxin A; DON: deoxynivalenol; 3ADON: 3-acetyl-deoxynivalenol; 15ADON: 15-acetyl-deoxynvalenol; D3G: deoxynivalenol-3-glucoside; T2: T-2 toxin; HT2: HT-2 toxin; FB1: fumonisin B1; FB2: fumonisin B2; FB3: fumonisin B3; ZEA: zearalenone; FA: fusaric acid; MPA: mycophenolic acid; PA: penicillic acid.

**Table 9:** Yearly variations in mean (SE) concentrations (µg/kg), and number of samples detected containing each mycotoxin group in corn grain (*n* = 711).

Mycotoxin Groups ^1^	Harvest Year							
	2013	2014	2015	2016	2017	2018	2019	*p*-Value
Concentrations, µg/kg								
Aflatoxins, total	2.3 (0.2)	2.9	4.1 (1.0)	3.1 (0.2)	310.1 (301.1)	4.7 (0.9)	19.2 (2.9)	<0.001
Ochratoxins	6.4 (0.2)	2.7 (0.5)	2.1 (0.03)	9.7	-	-	-	<0.001
Type B trichothecenes	437.3 (115.1)	897.0 (400.0)	283.1 (63.1)	799.3 (179.6)	1009.0 (163.7)	746.5 (73.0)	1673.4 (414.2)	<0.001
Type A trichothecenes	14.1 (3.4)	11.3 (4.0)	26.8 (6.5)	38.4 (26.6)	64.2 (12.9)	47.7 (15.4)	35.5 (11.3)	0.092
Fumonisins	2425.1 (297.3)	890.9 (382.3)	741.6 (185.4)	2734.2 (580.2)	4495.0 (1426.4)	7205.6 (593.3)	1268.8 (247.9)	<0.001
Zearalenone	243.0 (88.4)	93.8 (37.2)	15.8	344.4	446.6 (169.5)	402.6 (69.8)	228.7 (63.0)	<0.001
Emerging mycotoxins	327.7 (103.0)	471.7 (179.1)	71.5 (25.2)	140.7 (26.1)	284.3 (42.6)	158.6 (12.9)	225.6 (80.2)	<0.001
*Penicillium* mycotoxins	114.6 (53.5)	12.7 (10.9)	222.1 (93.4)	71.2 (39.1)	4.5 (1.0)	167.2 (57.1)	446.5 (297.8)	0.289
*Aspergillus* mycotoxins	5.3 (0.4)	2.5	-	-	-	334.0 (103.7)	39.6	<0.001
Ergot alkaloids	5.6 (0.9)	3.0 (0.8)	3.2 (0.6)	-	8.1	1.5 (0.1)	-	0.008
No. Positive Samples								
Aflatoxins, total	29	1	4	8	2	7	3	
Ochratoxins	29	6	12	1	0	0	0	
Type B trichothecenes	43	31	28	78	78	230	93	
Type A trichothecenes	15	2	5	6	13	41	12	
Fumonisins	40	30	35	57	46	222	63	
Zearalenone	10	11	1	1	10	59	59	
Emerging mycotoxins	40	31	22	62	63	252	85	
*Penicillium* mycotoxins	17	3	2	3	2	32	6	
*Aspergillus* mycotoxins	36	1	0	0	0	6	1	
Ergot alkaloids	9	4	5	0	1	4	0	

^1^ Aflatoxins, total: aflatoxin B1 + aflatoxin B2 + aflatoxin G1 + aflatoxin G2; ochratoxins: ochratoxin A + ochratoxin B; type B trichothecenes: deoxynivalenol + 3-acetyl-deoxynivalenol + 15-acetyl-deoxynvalenol + deoxynivalenol-3-glucoside + nivalenol + fusarenon X; type A trichothecenes: T-2 toxin + HT-2 toxin + diacetoxyscirpenol + neosolaniol; fumonisins: fumonisin B1 + fumonisin B2 + fumonisin B3; emerging mycotoxins: fusaric acid + alternariol; *Penicillium* mycotoxins: patulin + mycophenolic acid + roquefortine C + penicillic acid; *Aspergillus* mycotoxins: wortmannin + gliotoxin + sterigmatocystin + verruculogen; ergot alkaloids: ergometrine + ergotamine + ergocryptine + lysergol + methylergonovine.

**Table 10:** Yearly variations in mean (SE) concentrations (µg/kg), and number of samples detected containing each mycotoxin group in corn silage (*n* = 711).

	Harvest Year							
Mycotoxin Groups ^1^	2013	2014	2015	2016	2017	2018	2019	*p*-Value
Concentrations, µg/kg								
Aflatoxins, total	45.3 (10.8)	27.5	18.4 (2.6)	21.5 (4.6)	12.5 (2.5)	4.4 (0.5)	48.1 (23.3)	<0.001
Ochratoxins	4.8 (0.7)	4.6 (1.5)	3.2 (1.0)	7.0	42.4	36.7	12.2	0.023
Type B trichothecenes	1082.7 (189.4)	1974.7 (286.7)	1750.9 (251.5)	1440.7 (171.0)	2708.5 (266.3)	2353.7 (153.2)	1980.7 (192.1)	0.044
Type A trichothecenes	14.0 (3.1)	11.6 (4.9)	133.2 (35.0)	78.7 (30.9)	660.3 (138.8)	76.5 (9.5)	166.7 (34.2)	<0.001
Fumonisins	3405.3 (1367.0)	421.0 (82.1)	2625.1 (1035.6)	1530.2 (336.8)	2950.3 (596.9)	2540.8 (271.1)	1252.8 (324.7)	<0.001
Zearalenone	68.4 (19.2)	170.4 (30.4)	1253.9	1927.3	2233.2	964.7 (109.5)	452.8 (55.8)	<0.001
Emerging mycotoxins	1109.3 (173.5)	1778.4 (301.8)	478.0 (92.1)	378.3 (38.4)	997.1 (70.1)	415.2 (22.2)	702.1 (56.1)	<0.001
*Penicillium* mycotoxins	8.7 (1.6)	214.0 (145.9)	153.2 (20.3)	122.8 (46.9)	212.9 (68.9)	141.7 (15.4)	135.2 (49.8)	<0.001
*Aspergillus* mycotoxins	59.5 (21.7)	31.3	722.9	1.6	150.2	153.8 (70.1)	237.6	<0.001
Ergot alkaloids	12.8 (1.2)	10.7 (3.2)	10.5 (9.2)	5.4	11.7	11.4 (2.7)	209.9	<0.001
No. Positive Samples								
Aflatoxins, total	11	29	29	24	45	78	21	
Ochratoxins	6	8	2	4	2	1	1	
Type B trichothecenes	28	42	39	120	202	377	192	
Type A trichothecenes	20	3	12	18	50	163	57	
Fumonisins	25	35	31	74	146	308	133	
Zearalenone	10	25	1	1	1	55	106	
Emerging mycotoxins	25	43	41	120	209	396	214	
*Penicillium* mycotoxins	4	7	17	21	29	86	16	
*Aspergillus* mycotoxins	19	13	2	1	3	18	9	
Ergot alkaloids	8	17	5	40	49	100	7	

^1^ Aflatoxins, total: aflatoxin B1 + aflatoxin B2 + aflatoxin G1 + aflatoxin G2; ochratoxins: ochratoxin A + ochratoxin B; type B trichothecenes: deoxynivalenol + 3-acetyl-deoxynivalenol + 15-acetyl-deoxynvalenol + deoxynivalenol-3-glucoside + nivalenol + fusarenon X; type A trichothecenes: T-2 toxin + HT-2 toxin + diacetoxyscirpenol + neosolaniol; fumonisins: fumonisin B1 + fumonisin B2 + fumonisin B3; emerging mycotoxins: fusaric acid + alternariol; *Penicillium* mycotoxins: patulin + mycophenolic acid + roquefortine C + penicillic acid; *Aspergillus* mycotoxins: wortmannin + gliotoxin + sterigmatocystin + verruculogen; ergot alkaloids: ergometrine + ergotamine + ergocryptine + lysergol + methylergonovine.

**Table 11:** Distribution of samples by commodity, geographic origin, and year.

	Number of Samples (*n*)	Proportion (%)
Corn Grain		
Total number of samples	711	
Region		
Midwest	358	50.4
Northeast	201	28.3
Southeast	125	17.6
Southwest	14	2.0
Western	13	1.8
Harvest Year		
2013	51	7.2
2014	35	4.9
2015	48	6.8
2016	117	16.5
2017	83	11.7
2018	274	38.5
2019	103	14.5
Corn Silage		
Total number of samples	1117	
Region		
Midwest	539	48.3
Northeast	286	25.6
Southeast	69	6.2
Southwest	20	1.8
Western	203	18.2
Harvest Year		
2013	28	2.5
2014	46	4.1
2015	44	3.9
2016	158	14.1
2017	218	19.5
2018	405	36.3
2019	218	19.5

**Table 12:** Analyzed mycotoxins, lowest limit of detection or quantification by ultra-pressure liquid chromatography–tandem mass spectrometry.

	Mycotoxin Group	Mycotoxins	Abbreviation	Lowest Limit of Detection, µg/kg	Lowest Limit of Quantification, µg/kg
1	Total Aflatoxins	Aflatoxin B1	AFB1	0.129	0.429
2		Aflatoxin B2	AFB2	0.684	2.281
3		Aflatoxin G1	AFG1	0.449	1.495
4		Aflatoxin G2	AFG2	0.422	1.408
5	Ochratoxins	Ochratoxin A	OTA	0.362	1.208
6		Ochratoxin B	OTB	0.302	1.008
7	Type B Trichothecenes	Deoxynivalenol	DON	5.713	19.044
8		3-acetyl-deoxynivalenol	3ADON	4.058	13.526
9		15- acetyl-deoxynivalenol	15ADON	7.442	24.806
10		Deoxynivalenol-3-glucoside	D3G	16.651	55.50
11		Nivalenol	NIV	53.988	179.960
12		Fusarenon X	FX	2.489	8.295
13	Type A Trichothecenes	T-2 toxin	T2	0.744	2.481
14		HT-2 toxin	HT2	2.296	7.655
15		Diacetoxyscirpenol	DAS	1.505	5.017
16		Neosolaniol	NEO	0.946	3.154
17	Fumonisins	Fumonisin B1	FB1	20.426	68.086
18		Fumonisin B2	FB2	1.804	6.012
19		Fumonisin B3	FB3	2.918	16.493
20	Zearalenone	Zearalenone	ZEA	2.545	8.482
21	Emerging Mycotoxins	Fusaric acid	FA	0.017	0.055
22		Alternariol	ALT	1.379	4.598
23	*Penicillium* Mycotoxins	Patulin	PAT	16.669	55.562
24		Mycophenolic acid	MPA	2.496	8.319
25		Roquefortine C	ROQC	0.196	0.653
26		Penicillic acid	PEN	11.693	38.978
27	*Aspergillus* Mycotoxins	Wortmannin	WORT	0.764	2.545
28		Gliotoxin	GLIO	5.608	18.692
29		Sterigmatocystin	STERIG	0.184	0.612
30		Verruculogen	VER	0.331	1.104
31	Ergot Alkaloids	Ergometrine	ERGOM	0.573	1.911
32		Ergotamine	ERGOTA	0.502	1.673
33		Ergocryptine	ERGOCRP	0.803	2.677
34		Lysergol	LYSERG	0.457	1.522
35		Methylergonovine	MERGON	0.048	0.161

## Data Availability

Data will be provided upon request.
